# Fatal sarin poisoning in Syria 2013: forensic verification within an international laboratory network

**DOI:** 10.1007/s11419-017-0376-7

**Published:** 2017-07-21

**Authors:** Harald John, Marcel J. van der Schans, Marianne Koller, Helma E. T. Spruit, Franz Worek, Horst Thiermann, Daan Noort

**Affiliations:** 1 Bundeswehr Institute of Pharmacology and Toxicology, 80937 Munich, Germany; 2 Netherlands Organization for Applied Scientific Research TNO, 2280 AA Rijswijk, The Netherlands

**Keywords:** Biomarkers of exposure, LC–ESI-MS (/MS), Nerve agent, Organisation for the Prohibition of Chemical Weapons, Protein adducts, Verification analysis

## Abstract

During the United Nations fact-finding mission to investigate the alleged use of chemical warfare agents in the Syrian Arab Republic in 2013, numerous tissues from a deceased female victim, who had displayed symptoms of cholinergic crisis, were collected. The Organisation for the Prohibition of Chemical Weapons (OPCW) authorized two specialized laboratories in the Netherlands and Germany for forensic analysis of these samples. Diverse modern mass spectrometry (MS)-based procedures in combination with either liquid chromatography (LC) or gas chromatography (GC) separation were applied. A variety of biotransformation products of the nerve agent sarin was detected, including the hydrolysis product *O*-isopropyl methylphosphonic acid (IMPA) as well as covalent protein adducts with e.g., albumin and human butyrylcholinesterase (hBChE). IMPA was extracted after sample acidification by solid-phase extraction and directly analyzed by LC–tandem-MS with negative electrospray ionization (ESI). Protein adducts were found, either by fluoride-induced reactivation applying GC–MS techniques or by LC–MS-based detection after positive ESI for proteolyzed proteins yielding phosphonylated tyrosine residues or a specific phosphonylated hBChE-derived nonapeptide. These experimental results provided unambiguous evidence for a systemic intoxication and were the first proving the use of sarin in the ongoing bellicose conflict. This scenario underlines the requirement for qualified and specialized analytical laboratories to face repeated violation of the Chemical Weapons Convention.

## Introduction

Within the last decades to the present day, the use of chemical warfare agents (CWA) has been documented several times. CWA were deployed by state actors as well as terrorists, thus underlining a continuous threat to military and civilian personnel [1]. In spring and summer 2013, a number of alleged attacks with nerve agents took place in the Syrian Arab Republic during the ongoing conflict [2]. Deployment of CWA infringes upon the commonly accepted Chemical Weapons Convention, thus requiring qualified forensic analysis. During the concomitant United Nations fact-finding mission, biomedical samples were collected. Tissues of a dead female victim were taken several weeks after death. Immediately after poisoning on April 29, 2013 in the Syrian city of Saraqueb, the victim was reported to have shown miosis (contraction of the pupils) and other symptoms of cholinergic crisis, and died within 24 h after suspected exposure [2]. Exhibits from diverse organs, blood and hair were sent to the laboratory of the Organisation for the Prohibition of Chemical Weapons (OPCW, The Hague, The Netherlands, received Nobel Prize for Peace in 2013) in July 2013. Subsequently, samples were distributed to two laboratories in Germany (Bundeswehr Institute of Pharmacology and Toxicology, Munich) and The Netherlands (Netherlands Organization for Applied Scientific Research TNO, Rijswijk) for analysis [3]. The laboratories were asked to analyze the samples for the presence of signatures of anti-cholinesterase compounds, in particular those of sarin. The two laboratories did not know of each other's involvement, in order to guarantee fully independent analysis and reporting to the OPCW. Procedures applied were established individually in both laboratories.

**Fig. 1:**
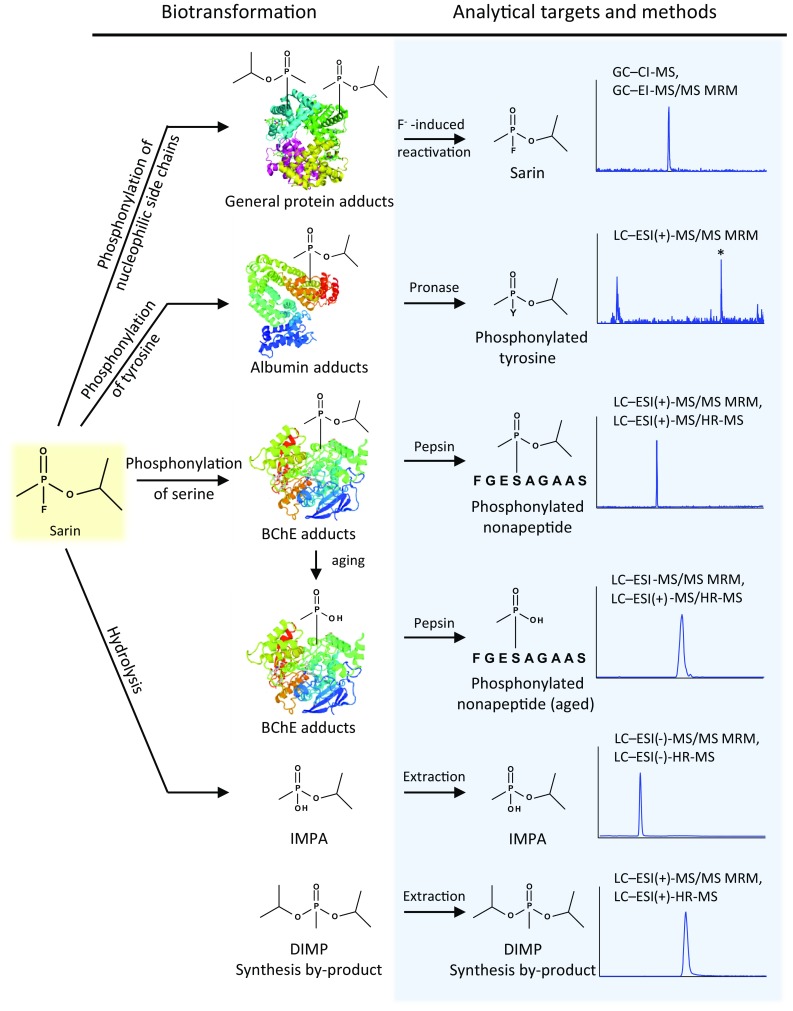
Biological fate of sarin and targets for biomedical verification of poisoning. Sarin undergoes two major biotransformation processes, i.e., hydrolysis and adduct formation. The resulting reaction products are unequivocal biomarkers of exposure, which can be assessed by modern mass spectrometric methods. *BChE* butyrylcholinesterase, *CI* chemical ionization, *DIMP* diisopropylmethylphosphonate, *EI* electron ionization, *ESI* electrospray ionization, *GC* gas chromatography, *HR* high resolution, *IMPA O*-isopropyl methylphosphonic acid, *LC* liquid chromatography, *MRM* multiple reaction monitoring, *MS* mass spectrometry, *MS/MS* tandem mass spectrometry

The limited stability and high reactivity of sarin (Fig. 1) precludes detection of the intact poison in vivo, thus requiring the search for more stable and long-lived surrogate parameters derived from biotransformation. The biological fate of sarin primarily consists of hydrolysis to *O*-isopropyl methylphosphonic acid (IMPA) (Fig. 1, 5th line). Additional transformation pathways comprise binding to acetylcholinesterase (AChE, EC. 3.1.1.7) and butyrylcholinesterase (BChE, EC 3.1.1.8; Fig. 1, 3rd line), albumin (Fig. 1, 2nd line) and various other less abundant proteins, thereby forming adducts (Fig. 1, 1st line) [4–6]. Adduct formation is based on a nucleophilic substitution of the leaving group of the nerve agent (fluoride) with the nucleophilic moiety of an amino acid side chain (e.g., hydroxyl moiety of serine in AChE and BChE or of tyrosine in albumin). The resulting modified protein thus contains the covalently attached phosphonyl moiety, whereas the leaving group is released (Fig. 1, 1st–4th lines). As typically observed as an in vivo phenomenon, the BChE adduct might undergo a degradation process called aging (Fig. 1, 4th line). During this reaction, the *O*-bound alkyl-group of the phosphonyl moiety is hydrolyzed, resulting in methylphosphonic acid (MPA) still attached to the protein (aged adduct). Accordingly, this product might also act as a diagnostic marker for poisoning with organophosphorus agents.

In contrast to hydrolysis products, protein adducts generally exhibit a much longer half-life in vivo, determined by the natural protein turnover (about 21 days for albumin and about 12 days for BChE) [6]. This long lifetime allows successful detection of poison incorporation even if samples could not be taken within a few days after exposure. Therefore, these targets represent highly valuable and specific biomarkers being indispensable for post-exposure analysis.

It should be noted that for both types of biomarkers the structure of the leaving group of the toxicant (F^−^ in case of sarin) is not revealed, which means that these biomarkers do not allow distinction among, e.g., sarin, chlorosarin or *O*-isopropyl VX (a VX analogue with an *O*-isopropyl moiety instead of an *O*-ethyl group). Nevertheless, both the hydrolysis product as well as the various protein adducts are generally accepted as unequivocal biomarkers for sarin exposure [6, 7]. Moreover, in any case, they all point to an organophosphorus agent belonging to the highly controlled class of OPCW schedule 1 chemicals [8], including toxic chemicals and their synthetic precursors.

## Materials and methods

### Chemicals and reagents

Acetonitrile (gradient grade), isopropanol (GC grade) and potassium fluoride (ACS grade) were purchased from Merck (Darmstadt, Germany); chloroform (≥99%), methanol (>99%, spectrophotometric grade), ethyl acetate (≥99%), NaHCO_3_ (ultra grade, ≥99.5%), *n*-hexane (≥99%) and pepsin (from pig gastric mucosa) from Sigma-Aldrich (Taufkirchen, Germany); pepsin (lot 12030521) and pronase (from *Streptomyces griseus*, lot no. 70327222) from Roche (Mannheim, Germany); NH_4_HCO_3_ (ultra grade, ≥99.5%) from Fluka (Buchs, Switzerland); formic acid (≥98%, ACS grade) and perchloric acid (70%) from Carl Roth (Karlsruhe, Germany); *O*-ethyl methylphosphonic acid (EMPA, 98%) and pinacolyl methylphosphonic acid (PMPA, 98%) from Aldrich (Milwaukee, WI, USA); *n*-butylphosphonic acid (nBPA, 98.5% NMR) from Lancaster (Eastgate, UK). Diisopropyl methylphosphonate (DIMP, 99% NMR), *O*-cyclohexyl methylphosphonic acid (cHMPA, 99% NMR), *O*-isobutyl methylphosphonic acid (iBMPA, 98.8% NMR), IMPA (98.5% NMR) and *O*-*n*-butyl methylphosphonic acid (nBMPA, 98.0% NMR) were produced by TNO (Rijswijk, The Netherlands); the syntheses based on a general protocol corresponding to Kranawetvogl et al. [9]. Isolute ENV+ cartridges for solid-phase extraction (SPE, 25 mg, 1 mL) were delivered by Biotage (Uppsala, Sweden); AbsElut-Nexus cartridges (200 mg) by Varian, Middelburg, The Netherlands; UF devices (molecular weight cut-off, MWCO, 10 kDa, Vivaspin 500 centrifugal concentrator) by Satorius Stedim (Göttingen, Germany) or 10 K Amicon Ultra-0.5 mL (UFC501096) by Merck.

### Tissue samples of poisoned victim

Tissue samples (blood, brain, breast fat, bronchus, eye, hair, heart, kidney, liver, lung, muscle and skin) from an anonymous dead woman who was supposed to be fatally poisoned by the nerve agent sarin were collected during the United Nations mission and forwarded to the OPCW. Samples were repackaged and transported independently to the Netherlands Organization for Applied Scientific Research, TNO (Rijswijk, The Netherlands) as well as the Bundeswehr Institute of Pharmacology and Toxicology (Munich, Germany) under escort of the OPCW laboratory chemists. This complete process was documented, and the chain of custody of all samples was maintained. Both laboratories analyzed the samples in parallel, targeting nerve agent biomarkers.

Human tissues (brain, fat, heart, kidney, liver, lung, muscle) used as blanks as well as to prepare positive control samples were obtained from dead bodies and provided by an institute of forensic medicine following ethics guidelines.

### Procedure I: detection of *O*-isopropyl methylphosphonic acid (IMPA)

Laboratories detected diverse biomarkers of sarin exposure using bioanalytical procedures described below (procedures I–IV).

IMPA was analyzed by tandem mass spectrometry (MS/MS) in multiple reaction monitoring (MRM) mode as well as by high-resolution mass spectrometry (HR-MS) without fragmentation following liquid chromatography (LC) separation and electrospray ionization (ESI) in negative mode, ESI (−).

#### LC–ESI(−)-MS/MS MRM

A 1200 LC system (Agilent Technologies, Waldbronn, Germany) delivered solvent A (0.5% v/v formic acid) and solvent B (acetonitrile) in gradient mode: time (min)/B (%) 0/0, 1/0, 2/20, 5/20, 7/80, 14/80, 15/0 and 21/0 including a 2-min equilibration period under starting conditions with a flow rate at 175 µL/min. Separation of 10 µL of sample was performed at 30 °C on a Hypercarb, carbon column (100 × 2.1-mm i.d., particle size 5 µm; Thermo Fisher Scientific, Dreieich, Germany) online coupled to an API5000 triple-quadrupole mass spectrometer (AB Sciex, Darmstadt, Germany). Detection was done in MRM mode after ESI (−) under the following conditions using nitrogen as the collision gas: ionization spray voltage −4500 V at 100 °C, curtain gas (CUR) 30 psi (2.07 × 10^5^ Pa), heater gas (GS1) 60 psi (4.14 × 10^5^ Pa), turbo ion spray gas (GS2) 40 psi (2.76 × 10^5^ Pa), declustering potential (DP) −40 V for IMPA, −30 V for nBPA, entrance potential (EP) −4 V, and cell exit potential (CXP) −11 V for IMPA (transition to *m/z* 94.6) and −13 V (transition to *m/z* 79.1). Transitions of deprotonated IMPA from *m/z* 136.9 to 94.6 (collision energy, CE −22 V) and from *m/z* 136.9 to 79.1 (CE −38 V) were monitored using unit resolution in the first and third quadrupole (Q1 and Q3) with a 150-ms dwell time. nBPA was used as an internal standard that was recorded with transitions from *m/z* 137.1 to 79.1 (CE −30 V, EP −4 V, CXP −11 V) and to *m/z* 62.7 (CE −74 V, EP −4 V, CXP −11 V). MS data analysis and control of the mass spectrometer was done with Analyst 1.6.1 software (AB Sciex).

#### LC–ESI(−)-HR-MS

A Thermo Scientific Ultimate 3000 high-performance liquid chromatography (HPLC) system was used, with an injection volume of 10 µL. Solvent A (0.2% v/v formic acid in water) and solvent B (0.2% v/v formic acid in acetonitrile) were used in gradient mode as follows: time (min)/B (%) 0/0 and 30/45 in 30 min with a flow rate at 80 µL/min. A PepMap100 C18 column (150 × 1.0-mm i.d., particle size 3 µm, Thermo Fisher Scientific) was used at ambient temperature. The HPLC system was connected to a Maxis Impact quadrupole time-of-flight (QTOF) mass spectrometer (Bruker Daltonics, Bremen, Germany) operating with ESI (−) at −3000 V in the range of *m/z* 40–1400 at a resolution of 20,000.

#### Sample preparations

Blood: blood appeared as a black suspension, not allowing separation of a plasma fraction. Therefore, the sample was subjected to preparation steps as it was. For detection of IMPA, 0.5 mL of blood was treated as described below for the supernatant of organ homogenates.

Hair: minced hair (69 mg) was extracted with a mixture (1 mL) of 0.1% w/v aqueous NH_3_/methanol (50:50 v/v) for 90 min at ambient temperature. Afterwards, the liquid phase was evaporated to remove methanol and NH_3_, and mixed with 2% (v/v) formic acid (0.5 mL) to restore a total volume of 1 mL ready for LC–ESI(−)-MS/MS MRM analysis of IMPA.

Organ tissues: diverse tissues from brain, breast fat, bronchus, eye, heart, kidney, liver, lung, muscle and skin were prepared following a common protocol including homogenization, centrifugation and SPE of the supernatant. Initially weighted wet tissue was added to a defined volume of water (see below) prior to homogenization using an Ultra Turrax (IKA, Staufen, Germany) equipped with a disposable knife for 5 cycles of 10 s each under ice cooling (13,500 rpm): brain (1.153 g/3.45 mL water), breast fat (0.987 g/2.97 mL water), bronchus (1.88 g/5.64 mL water), eye (2.783 g/8.34 mL water), heart (2.181 g/6.54 mL water), kidney (2.148 g/6.44 mL water), liver (2.312 g/6.94 mL water), lung (2.614 g/7.83 mL water), muscle (1.689 g/5.07 mL water) and skin (1.014 g/3.05 mL water). Aliquots of the homogenates (0.5 mL) were stored at −80 °C if not prepared immediately. Aliquots were mixed with 10 µL of an internal standard solution (nBPA in methanol, 1 µg/mL) prior to addition of 1 M perchloric acid (150 µL) for protein precipitation. After centrifugation (20,000 rpm, 10 min, 4 °C) the supernatant was subjected to SPE on Isolute ENV+ (25 mg) preconditioned with methanol and water (1 mL each). The cartridge was rinsed with 250 µL of water und sucked dry for 5 min prior to elution using a mixture of 0.1% w/v aqueous NH_3_ and methanol (50:50 v/v). Afterwards, the eluate was evaporated to remove methanol and NH_3_ and mixed with 2% (v/v) formic acid (0.5 mL) to restore a total volume of 1 mL ready for LC–ESI(−)-MS/MS MRM analysis of IMPA.

Positive control samples: blank tissues were homogenized (33 mg wet weight/mL water) as described above and spiked with a mixture of diverse methylphosphonic acids including cHMPA, EMPA, iBMPA, IMPA, nBMPA, PMPA and nBPA (10 ng/mL each) prior to further sample preparation following the described protocol.

### Procedure II: fluoride-induced reactivation of protein-bound sarin

Two GC-based methods were used following either MRM or full-scan technique for MS detection in electron ionization (EI) and chemical ionization (CI) modes.

#### GC–EI-MS/MS MRM

A Trace GC Ultra instrument (Thermo Fisher Scientific) was used with helium as carrier gas, in programmable temperature vaporization splitless injection mode at a flow rate of 1.5 mL/min. Injector temperature was 250 °C and a volume of 1 µL was injected. The instrument was equipped with a J&W VF-5MS column (45 m × 0.32-mm i.d., film thickness 0.4 µm, Agilent Technologies). The GC temperature program was as follows: time (min)/oven temperature (°C) 0/40, 1/40, 10/130, 15/280 and 20/280. The GC was coupled to a TSQ Quantum mass spectrometer (Thermo Fisher Scientific) operating in MRM mode in positive polarity with an electron energy of 70 eV. The solvent delay time was 4 min. Precursor ions were fragmented using argon as collision gas and a collision energy of 10 eV with monitoring transitions from *m/z* 125 to 99 and from *m/z* 99 to 81.

#### GC–CI-MS

A 6890 Series II GC system (Agilent Technologies) equipped with an injector from Gerstel (Mühlheim, Germany) was used with helium as carrier gas, in splitless injection mode (splitless time for 0.75 min) with a constant flow at 1.5 mL/min. The injector temperature was 250 °C, and 1 µL was injected. The instrument was equipped with a J&W VF-5MS column (50 m × 0.32-mm i.d., film thickness 0.4 µm, Agilent Technologies). The GC temperature program was as follows: time (min)/oven temperature (°C) 0/40, 1/40, 10/130, 15/280 and 20/280. The GC was connected to a 5973 N mass spectrometer (Agilent Technologies) operating in selected ion monitoring mode (*m/z* 158) with positive polarity and an ionization energy at 235 eV using ammonia as reaction gas (flow 21%). The solvent delay time was 4 min. Source temperature was set to 160 °C.

#### Sample preparations

Organ tissue: the fluoride reactivation method was performed according to the method published by Holland et al. [10]. A 25% (w/v) tissue homogenate in water was prepared from 1 g of tissue. A portion of 1 mL of homogenate was diluted with 3 mL 0.189 M acetate buffer (pH 3.4). Potassium fluoride was added (190 µL of 5.25 M) to a final concentration of 0.25 mM. The fluoride reactivation reaction was allowed for 15 min at 25 °C. Next, 500 µL of 0.8 M NaHCO_3_ was added and the mixture was applied on a pre-conditioned AbsElut-Nexus cartridge (200 mg). Preconditioning of the AbsElut-Nexus cartridge implied consecutive rinsing with 1 × 4 mL of *n*-hexane, 2 × 4 mL of ethyl acetate and 1 × 5 mL of water. The cartridge was rinsed with 5 mL of water and dried with air. The fluoridated compound was eluted with 2 mL of chloroform. A volume of 100 µL of ethyl acetate was added and the solvent was evaporated to a final volume of approx. 100 µL. The resulting solution was used for GC–MS analyses.

Blood: blood (500 µL) was diluted with 3 mL of 0.189 M acetate buffer (pH 3.4). Potassium fluoride was added (190 µL of 5.25 M) to a final concentration of 0.25 mM. The fluoride reactivation reaction was allowed for 15 min at 25 °C. Next, 500 µL of 0.8 M NaHCO_3_ was added and the mixture was applied on a pre-conditioned AbsElut-Nexus Cartridge (200 mg). Preconditioning of the AbsElut-Nexus cartridge implied consecutive rinsing with 1 × 4 mL of *n*-hexane, 2 × 4 mL of ethyl acetate and 1 × 5 mL of water. The cartridge was rinsed with 5 mL water and dried with air. The fluoridated compound was eluted with 2 mL of chloroform. A volume of 150 µL of ethyl acetate was added and the solvent was evaporated to a final volume of approximately 150 µL. The resulting solution was used for GC–MS analyses. A blank blood sample was processed in the same way.

### Procedure III: analysis of human butyrylcholinesterase (hBChE) adducts

Human BChE adducts were analyzed by MS in MRM mode as well as by HR-MS without fragmentation following LC separation.

#### LC–ESI(+)-MS/MS MRM

An Acquity HPLC system (Waters, Etten-Leur, The Netherlands) was used, employing an injection volume of 10 µL. The eluent consisted of solvent A (0.2% v/v formic acid in water) and solvent B (0.2% v/v formic acid in acetonitrile). The elution program was as follows: time (min)/B (%) 0/0 and 20/80 with a flow at 100 µL/min. An Acquity HSS T3 column (100 × 2.1-mm i.d., particle size 1.8 µm, Waters) was used at ambient temperature. The HPLC was connected to a TSQ Quantum Ultra mass spectrometer (Thermo Fisher Scientific) with ESI (+) at 3000 V. The MRM mode detected transitions from *m/z* 874 (aged nonapeptide adduct of sarin) to 602, 673 and 778 using collision energies of 31, 27 and 26 V, respectively.

#### LC–ESI(+)-MS/HR-MS

A Thermo Scientific Ultimate 3000 HPLC system was used, with an injection volume of 10 µL. Solvent A (0.2% v/v formic acid in water) and solvent B (0.2% v/v formic acid in acetonitrile) were used in gradient mode as follows: time (min)/B (%) 0/0 and 30/45 with a flow at 80 µL/min. A PepMap100 C18 column (150 × 1.0-mm i.d., particle size 3 µm, Thermo Fisher Scientific) was used at ambient temperature. The HPLC system was connected to a Maxis Impact QTOF mass spectrometer (Bruker Daltonics) operating with ESI (+) at 4000 V. Product ions of the sarin-adducted nonapeptide (*m/z* 916) and its aged variant (*m/z* 874) were recorded in the full-scan MS/MS mode in the range from *m/z* 200 to 1550 applying a collision energy of 35 V.

#### Sample preparation

Phosphylated hBChE was isolated according to the method using immunomagnetic separation as published by Sporty et al. [11]. Blood (200 µL) was incubated with 25 µL of hBChE antibody-coated magnetic beads for 2 h. Beads were washed with phosphate-buffered saline, suspended in water and recovered from water. Next, the beads were incubated in 75 µL of pepsin solution (0.25 mg/mL pepsin in 0.63% v/v formic acid) for 1.5 h at 37 °C. The liquid containing the pepsin digest was ultrafiltrated through a 10-kD MWCO filter (10 K Amicon Ultra, 0.5 mL, UFC 501096, Merck, Z 677108-96EA). Next, the filter was rinsed with additional 100 µL of 0.63% v/v formic acid, and filtrates were pooled. A blank blood sample was processed in the same way. Samples were analyzed by LC–ESI(+)-MS/MS in MRM mode as well as by LC–ESI(+)-MS/HR-MS to detect the intact adducted nonapeptide and its aged variant with injecting 10 µL each.

### Procedure IV: analysis of tyrosine adducts

#### Micro LC–ESI(+)-MS/MS MRM

The micro LC system consisted of a 1431 MicroPro pump (Eldex Laboratories, Napa, CA, USA), an Endurance autosampler (Spark Holland, Emmen, The Netherlands), a Mistral column oven (Spark Holland), a SpectraFlow 2020 UV/Vis detector (Sunchrom, Friedrichsdorf, Germany) and a Degasys Populaire degasser (Sunchrom). Pumps were controlled by MicroPro 1.0 software (SCPA, Weyhe-Leeste, Germany), and the autosampler by Endurance/Midas 3.10 (SCPA). Solvent A (0.05% v/v formic acid) and solvent B (acetonitrile/water 80:20, 0.05% v/v formic acid) were used in gradient mode as follows: time (min)/B (%) 0/0, 5/0, 38/40, 39/80, 44/80 and 45/0 after a 30-min equilibration period under starting conditions. Separation was carried out at 30 °C on an Acclaim PepMap 100, C18 (150 × 1.0-mm i.d., particle size 3 µm, 100 A, Thermo Fisher Scientific) connected with a precolumn (security guard cartridges, widepore C18 4 × 2-mm i.d., Phenomenex, Aschaffenburg, Germany) with a flow of 20 µL/min. Online mass spectrometric detection was performed using an API 4000 QTrap triple quadrupole system (AB Sciex, Darmstadt, Germany) with ESI (+) applying nitrogen as the collision gas. MS parameters were as follows: IS +3500 V at 300 °C, CUR 30 psi (2.07 × 10^5^ Pa), GS1 and GS2 50 psi (3.45 × 10^5^ Pa), DP 60 V, EP 10 V and CXP 10 V. The protonated tyrosine-sarin adduct (*m/z* 302.1) was monitored by selective transitions to *m/z* 260, 214.0, 197 and 136.0, all produced with a CE of 25 V using unit resolution for Q1 and Q3 with a 100-ms dwell time. MS data analysis and control of the mass spectrometer was done with Analyst 1.6 software (AB Sciex).

#### Sample preparation

For detection of the tyrosine adduct, 150 µL of blood was centrifuged (18,600×*g*, 7 min, 15 °C) before transferring the supernatant (100 µL) into an ultrafiltration (UF) device (10 kDa cut-off). The sample was washed three times by UF (10,300×*g*, 7 min, 15 °C) after addition of 400 µL of 50 mM NH_4_HCO_3_ (pH 8.0). Subsequently, 100 µL of pronase solution (10 mg/mL in 50 mM NH_4_HCO_3_) and 50 µL of NH_4_HCO_3_ buffer were added to the retentate (100 µL) followed by a 9-h incubation under gentle shaking at 37 °C. Afterwards, the mixture was ultrafiltrated again. The retentate was mixed with 10% v/v formic acid (100 µL) prior to a final UF step. Combined filtrates (250 µL) were subjected to micro LC–ESI(+)-MS/MS MRM analysis.

### Procedure V: analysis of diisopropylmethylphosponate (DIMP)

DIMP was detected by MRM and HR-MS technique after LC separation.

#### LC–ESI(+)-MS/MS MRM

The LC–ESI(+)-MS/MS system including column, solvents and gradient were the same as described above for analysis of hBChE adducts (procedure III). The MRM mode was employed with transitions from *m/z* 181 to 139 and 97, and from *m/z* 139 to 97, 79 and 47. Ionization voltage was 3000 V and collision energy 9 V.

#### LC–ESI(+)-HR-MS

The LC–ESI(+)-HR-MS system including column and solvents were the same as described above for analysis of hBChE adducts (procedure III). The gradient was as follows: time (min)/B (%) 0/0 and 30/80 with a flow at 80 µL/min. The HPLC system was connected to the Bruker Maxis Impact Q-TOF mass spectrometer operating with positive ESI (4000 V). Full-scan mass spectra were acquired at a resolution of 13,000 in the range *m/z* 40–1250.

#### Sample preparation

Representative extraction procedure (from the skin): to a piece of the skin (1.89 mg) without fat, 500 µL of 0.2% v/v formic acid in water was added, and the mixture was sonicated for 5 min. The extract was ultrafiltrated using a 10-kD MWCO filter (12,000 rpm, 25 min). The filtrate was analyzed.

## Results and discussion

A number of validated analytical procedures, with sufficient sensitivity to analyze the biomarkers in the sub-nanomolar range, were applied by both laboratories in order to identify signatures of nerve agents, with special focus on sarin (Fig. 1). As most methods were primarily designed for analysis of human plasma, sample preparation procedures had to be adapted to the relevant tissues. According to the requirements given by the OPCW, qualitative analysis was sufficient for verification of exposure. Therefore, applicability of chosen procedures was demonstrated by positive control samples generated from each corresponding blank tissue incubated with sarin as well as by tissue-specific blank samples characterizing the absence of potential interferences. The four most relevant procedures are outlined in the following.

Procedure I, analysis of IMPA: a robust method for analysis of the hydrolysis product of sarin involved acid-promoted extraction from the aqueous matrix of a homogenized tissue sample either by SPE or liquid-liquid extraction followed by LC–ESI(−)-MS/MS MRM, by LC–ESI(−)-HR-MS or by GC–EI-MS after derivatization [7, 12] (Fig. 1, 5th line). It could be expected that in an exposed victim who survived, the plasma levels of hydrolysis products would rapidly decrease, because of normal excretion into urine. However, in this case, the victim had deceased 24 h after the exposure, and, therefore, we envisaged that significant levels of IMPA should be present.

Procedure II, fluoride-induced reactivation of protein-bound sarin: protein adducts formed by sarin-mediated phosphorylation are prone to be reactivated by nucleophiles substituting the bound amino acid side chain. Therefore, biological samples were treated with a large excess of fluoride ion in order to regenerate bound sarin by reversing the binding reaction of the active agent [13]. The resulting phosphonofluoride could subsequently be isolated by means of SPE and analyzed by GC–EI-MS/MS MRM (Fig. 1, 1st line). In this way, very low cholinesterase inhibition levels (down to 0.1%) could be monitored [10], that were even far below toxicologically relevant exposure levels. In contrast to unaltered phosphonyl moieties, aged adducts were not prone to this regeneration process due to the negatively charged methylphosphonic acid, hindering a nucleophilic attack [6].

Procedure III, analysis of hBChE adducts: a more generic method for organophosphate adduct analysis comprises the pepsin-mediated cleavage of the human protein BChE, isolated from the various tissues through an immunomagnetic method based on Sporty et al. [11]. The enzymatic cleavage of phosphonylated hBChE resulted in the nonapeptide FGES*AGAAS (with S* the active site serine residue bearing the particular modification; Fig. 1, 3rd line). This modified peptide undergoes fragmentation in the MS/MS mode producing three characteristic product ions [11, 14, 15]. The resulting fragment ions allowed reliable and selective identification of the nerve agent adduct. Aged hBChE adducts could be detected accordingly (Fig. 1, 4th line).

Procedure IV, analysis of tyrosine adducts: this method is based on pronase-catalyzed proteolysis of the entire blood sample that had undergone biological degradation during the weeks after death [16]. This procedure liberated most presumably the phosphonylated tyrosine-411 residue from human serum albumin, but may also produce the corresponding tyrosine adduct derived from other proteins or proteinaceous breakdown products [4] (Fig. 1, 2nd line). The adducted tyrosine was selectively detected after collision-induced dissociation (CID) of the protonated precursor ion in LC–ESI(+)-MS/MS MRM analysis. The advantage of this method was that aging reactions usually not readily occurred, thereby preserving more precise information of the actual nerve agent.

**Fig. 2:**
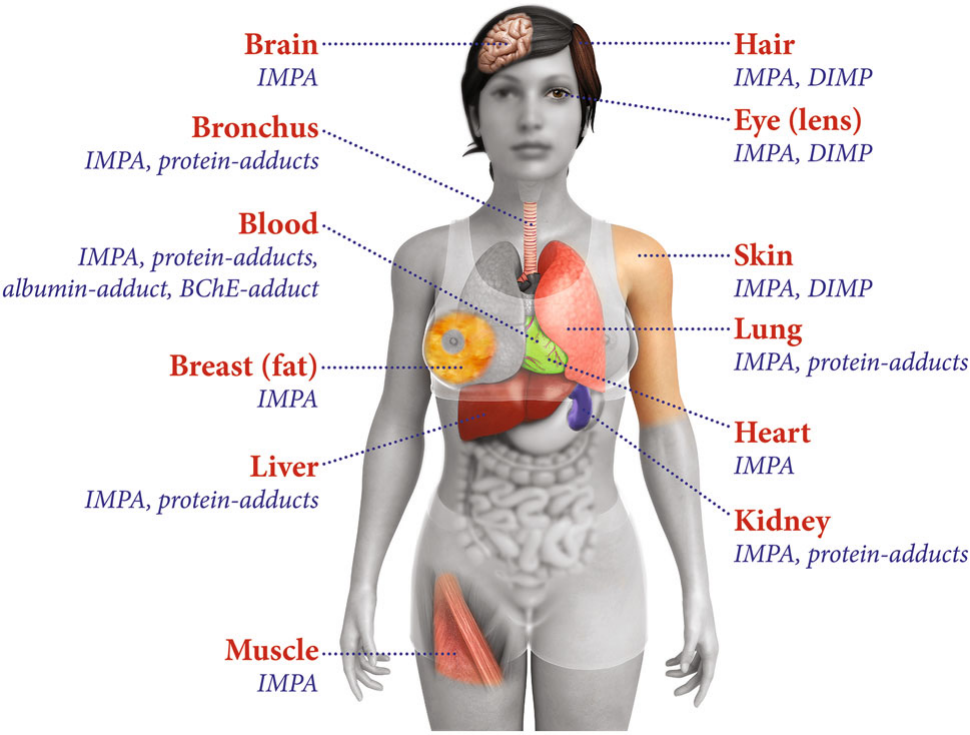
Analyzed organs of a female victim of sarin exposure and detected biomarkers of poisoning. Detection of poison markers by using diverse bioanalytical mass spectrometric techniques revealed systemic distribution of the nerve agent sarin. Tissues were analyzed by specialized German and Dutch laboratories on behalf of the Organisation for the Prohibition of Chemical Weapons (OPCW). Protein adducts in general are covalent reaction products resulting from phosphonylation of nucleophilic side-chains of amino acids by sarin, e.g., albumin and BChE adducts. Adducts are suitable long-term biomarkers of exposure

Described procedures allowed unambiguous selective detection of biomarkers indicating poisoning by the nerve agent sarin. Figure 2 graphically summarizes which biomarker of sarin exposure was found in the diverse tissue exhibits. The hydrolysis product IMPA was found in nearly all tissues, indicating systemic distribution of the intact poison. This finding is in line with the fact that the victim had deceased shortly after exposure, preventing excretion of IMPA via urine. Therefore, it is conceivable that the polar hydrolysis product remained in the various target tissues. Diffusion into other tissues due to postmortem processes also might have contributed to this dispersal.

**Fig. 3:**
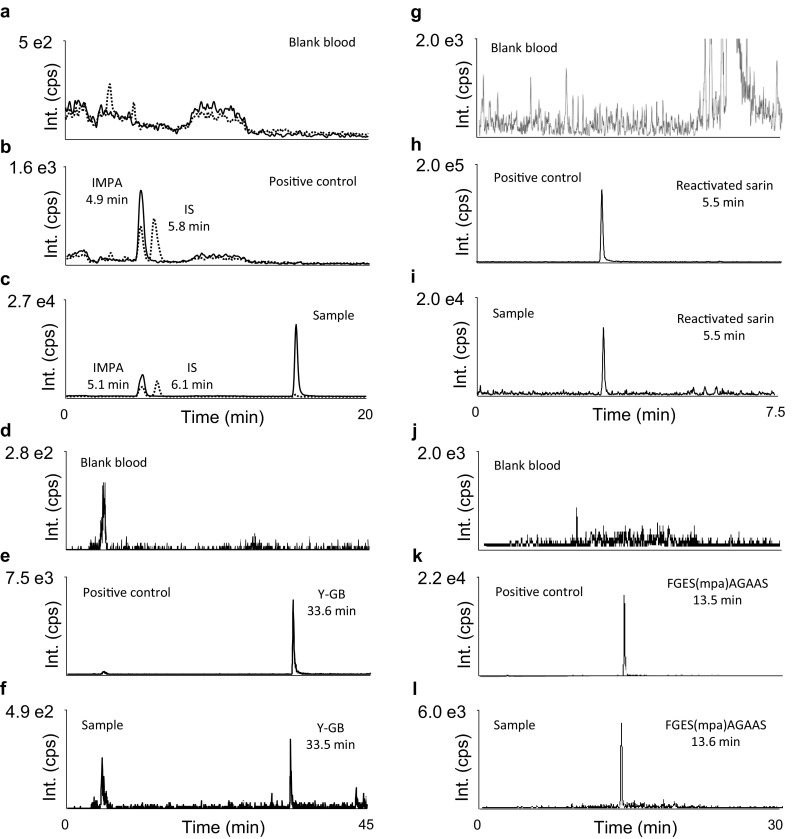
Analysis of a blood sample proving incorporation of the nerve agent sarin by diverse biomarkers. **a**–**c** Detection of hydrolyzed sarin (IMPA) by LC–ESI(−)-MS/MS MRM (procedure I) in a positive control (**b**) and the sample (**c**). For reasons of clarity, only two transitions for monitoring deprotonated IMPA (from *m/z* 136.9 to 94.6, *solid line*) and the internal standard (IS) *n*-butylphosphonic acid (nBPA, from *m/z* 137.1 to 79.1, *dotted line*) are depicted. **d**–**f** Detection of tyrosine adduct (Y-GB), most presumably derived from albumin. Analysis was performed by micro LC–ESI(+)-MS/MS MRM (procedure IV). Transition from *m/z* 302.1 to 214.1 is depicted exemplarily for a blank sample (**d**), positive control (**e**) and the sample (**f**). **g**–**i** Detection of sarin after fluoride-induced reactivation by GC–EI-MS/MS MRM (procedure II). Transition from *m/z* 125 to 99 is illustrated for blank (**g**), positive control (**h**) and sample (**i**). **j**–**l** Detection of the modified nonapeptide FGESAGAAS derived from the aged adduct of butyrylcholinesterase and sarin. Analysis was carried out by LC–ESI(+)-MS/MS MRM (procedure III). Transition from *m/z* 874 to 778 is shown for measurement of blank (**j**), positive control (**k**) and sample (**l**). Blank blood was free of interferences for each method (**a**, **d**, **g**, **j**)

Representative chromatograms for the analysis of the victim´s blood are shown in Fig. 3, illustrating detection of IMPA (Fig. 3c), F^−^-reactivated sarin (Fig. 3i), tyrosine adduct (Y-GB) most presumably derived from albumin (UniProt acc. No P02768) (Fig. 3f), and aged human BChE adduct (Fig. 3l).

Surprisingly, the intact adduct of sarin to hBChE was not observed, but instead the aged adduct with MPA was detected exclusively (Figs. 2, 3l). This suggests that aging happened either quite rapidly in the victim within 24 h, or occurred through postmortem processes after death before sample taking. For comparison, when we analyzed the plasma samples of Japanese victims of the Tokyo Subway sarin attack, the expected *O*-isopropyl methylphosphonic BChE adduct was found [12]. We, therefore, consider the postmortem aging reaction as the most likely explanation for the presence of the MPA-BChE adduct.

After applying the fluoride-induced reactivation method to blood (Fig. 3i) and several tissue samples, regenerated sarin derived from protein adducts could be analyzed as illustrated in Fig. 2. The fluoride-induced reactivation method was also applied on muscle, brain and heart tissue, but regenerated sarin was not detected. Therefore, the regenerated sarin obviously could not result from the hBChE adducts, but is apparently stemming from additional binding sites in other proteins that are not or less prone to the aging reaction [6]. Binding sites additional to hBChE indicate that the level of sarin exposure had been quite high, inducing saturation of binding sites in cholinesterases and making excess agent available for binding to other proteins. Adducts formed with albumin may represent a reasonable source for bound sarin, but a number of additional tissue proteins might also contribute to that positive finding. This is in line with the fact that the adduct of sarin to tyrosine was detected in blood sample(s), as presented in Fig. 3f, which is probably the result from phosphonylation of residue 411 or additional, less reactive Tyr residues (e.g., #148, 150 or 161) in human serum albumin [17].

**Fig. 4:**
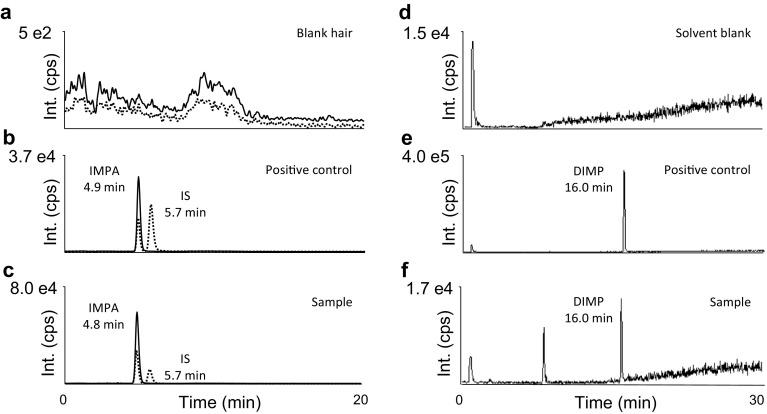
Analysis of a hair sample proving exposure to the nerve agent sarin. **a**–**c** Detection of hydrolyzed sarin (IMPA) by LC–ESI(−)-MS/MS MRM (procedure I) in a positive control (**b**) and the sample (**c**). For reasons of clarity, only two transitions for monitoring deprotonated IMPA (from *m/z* 136.9 to 94.6, *solid line*) and the IS (nBPA) (from *m/z* 137.1 to 79.1, *dotted line*) are depicted. **d**–**f** Detection of DIMP, a by-product of sarin synthesis, by LC–ESI(+)-HR-MS (*m/z* 97.005) is illustrated for a blank (**d**), positive control (**e**) and sample (**f**). Blank hair (**a**) and solvent blank (**d**) were free of interferences

In addition, in a number of samples (hair, skin), DIMP (Fig. 1, 6th line), one of the most common contaminants formed during production of sarin [18], was detected by LC–ESI(+)-MS/MS MRM and LC–ESI(+)-HR-MS (Figs. 2, 4f). The presence of this additional sarin signature further corroborates the forensic evidence based on the true sarin biomarkers (i.e., the hydrolysis product and protein adducts).

## Conclusions

In conclusion, this is the first report providing a comprehensive set of bioanalytical data obtained from numerous tissues of one victim that proved a real case of human poisoning by sarin. A high grade of evidence was achieved by combining diverse methods providing congruent results obtained by both laboratories mandated (Fig. 1).

Meanwhile, the OPCW has fully embraced the analysis of biomedical samples in cases of investigations for an alleged use of chemical warfare agents. Since 2016, annual biomedical proficiency tests (BioPT) are being held to designate laboratories qualified for biomedical verification analysis. According to the OPCW guidelines, quality criteria for sufficient evidence of a warfare agent-derived analyte are dictated. The herein presented analytical results meet these strict criteria for most of the samples, thus underlining analytical quality and autonomy of the involved laboratories. Since early 2016, both the Dutch and German laboratories have been officially designated by OPCW for analysis of biomedical samples for investigations of alleged use of chemical warfare agents.
